# Is Hip Muscle Strength Associated with Dynamic Knee Valgus in a Healthy Adult Population? A Systematic Review

**DOI:** 10.3390/ijerph18147669

**Published:** 2021-07-19

**Authors:** Ali Mohammed Alzahrani, Msaad Alzhrani, Saeed Nasser Alshahrani, Wael Alghamdi, Mazen Alqahtani, Hosam Alzahrani

**Affiliations:** 1 Almandq General Hospital, Ministry of Health, Albaha 65749, Saudi Arabia; 2 Department of Physical Therapy and Health Rehabilitation, College of Applied Medical Sciences, Majmaah University, Al Majmaah 11952, Saudi Arabia; m.alzhrani@mu.edu.sa (M.A.); mm.alqahtani@mu.edu.sa (M.A.); 3 Armed Forces Medical Services, Armed Forces Hospital, Southern Region, Khamis Mushait 61961, Saudi Arabia; alzaben.15@hotmail.com; 4 Department of Nursing, Faculty of Applied Medical Sciences, Albaha University, Albaha 65511, Saudi Arabia; waelalghamdi@bu.edu.sa; 5 Department of Physical Therapy, College of Applied Medical Sciences, Taif University, Taif 21944, Saudi Arabia; halzahrani@tu.edu.sa

**Keywords:** dynamic knee valgus, dynamic lower extremity valgus, hip strength, biomechanics, 2D motion analysis

## Abstract

This study aimed to systematically review research investigating the association between hip muscle strength and dynamic knee valgus (DKV). Four databases (MEDLINE, PubMed, CINAHL, and SPORTDiscus) were searched for journal articles published from inception to October 2020. Seven studies investigating the association between hip muscle strength and DKV using a two-dimensional motion analysis system in healthy adults were included. The relationship between hip abductor muscle strength and DKV was negatively correlated in two studies, positively correlated in two studies, and not correlated in three studies. The DKV was associated with reduced hip extensor muscle strength in two studies and reduced hip external rotator muscle strength in two studies, while no correlation was found in three and five studies for each muscle group, respectively. The relationship between hip muscle strength, including abductors, extensors, and external rotators and DKV is conflicting. Considering the current literature limitations and variable methodological approaches used among studies, the clinical relevance of such findings should be interpreted cautiously. Therefore, future studies are recommended to measure the eccentric strength of hip muscles, resembling muscular movement during landing. Furthermore, high-demand and sufficiently challenging functional tasks revealing lower limb kinematic differences, such as cutting and jumping tasks, are recommended for measuring the DKV.

## 1. Introduction

Knee valgus malalignment is a common dysfunction observed in the lower extremity during dynamic activities and has been suggested to be an underlying mechanism of knee injury [1,2,3]. Malalignment in the knee valgus usually occurs when there is simultaneous adduction and internal rotation of the femur on the tibia during closed-chain knee flexion [4]. There is a growing body of evidence supporting the influence of impaired hip control, as well as changes in lower limb mechanics, on the knee joint, which may contribute to injuries [5,6].

The hip plays an integral role in maintaining balance and providing stability, as the proximal articulation, for the lower limbs [7]. However, this functionality relies on a multifaceted interaction of hip muscles (e.g., hip abductor, external rotator, and extensor) to offer dynamic stability during motion [8]. Hence, hip muscle weakness may result in certain movement dysfunctions that can place certain muscles and joints, particularly the knee, in positions that are deemed to be at high risk of injury [5,6]. Furthermore, weakness of muscles surrounding the thigh and hip is thought to be an underlying mechanism for excessive knee valgus motion during dynamic movements, especially during challenging tasks such as ballistics and squats [9].

A substantial body of research has investigated the relationship between hip muscle strength and dynamic knee valgus (DKV). Two similar reviews have investigated the relationship and established limited evidence, alongside conflicting findings among studies [10,11]. The review by Cashman [10] failed to reach a definitive conclusion, primarily due to the variation in methodology and lack of consensus among the studies considered for the review. However, this review included studies published up to February 2011. As such, new knowledge has emerged regarding recent studies that have been undertaken. Another meta-analysis published by Dix et al. [11] established that a reduction in the strength of hip extensors, external rotators, and abductors is associated with the DKV during single-leg drop landings, but not double-leg landing. They suggested that the distinction in the results of the kinematic evaluation of the lower limbs between single- and double-leg tasks is largely due to varying demands and muscle recruitments that require significant eccentric work to provide control in the frontal plane angles. However, this review included female participants only (thus cannot be generalized) and injured participants. Including injured participants could confound the association between hip muscle strength and DKV because injuries and pain (e.g., knee osteoarthritis, patellofemoral pain, and some other lower extremity injuries) can cause DKV [12,13,14]; therefore, studies are not able to differentiate whether muscle weakness is the cause of the DKV or whether it occurs due to some mechanism of compensation or inhibition by the body in response to injury or pain. Furthermore, the review by Dix et al. [11] included studies that only utilized three-dimensional (3D) motion analysis in the measurement. It is worth noting that the two-dimensional (2D) motion system, however, has been proven to be an easier and more clinically accessible and applicable motion analysis approach compared to the 3D system, which requires expensive and laboratory-based procedures [15]. Furthermore, 2D motion analysis has been reported to have high reliability and adequate validity compared to 3D motion analysis [16,17,18].

This systematic review aimed to synthesize the current evidence investigating the relationship between hip muscle strength (abductor, extensor, or external rotator) and DKV during dynamic tasks among healthy adults using a 2D analysis system.

## 2. Methods

### 2.1. Eligibility Criteria

Observational peer-reviewed studies were included if they investigated the association between hip muscle strength and DKV in healthy adults of both sexes (aged ≥ 18 years) using 2D analysis systems. Eligible studies had no distinct time frames for the testing procedure. Literature reviews, case reports, and non-English articles, as well as studies recruiting participants with a pathology or previous history of injuries, were excluded.

### 2.2. Study Search Process

The search strategy was conducted following the guidelines published by the Preferred Reporting Items for Systematic Review and Meta-analysis protocols (PRISMA-P) [19]. This strategy was structured and developed according to the population (P), intervention (I), comparator (C), and outcome (O) (PICO) framework, as indicated by the PRISMA guidelines [19]. Using the PICO criteria, the following question was formulated: Is there a relationship between hip muscle strength and DKV during dynamic tasks among healthy adults using a 2D analysis system? For example, P—healthy adults, I—hip muscle strength, C—counterpart, and O—the DKV during dynamic tasks measured using 2D analysis system.

The following databases were employed for the search process on July 2018: MEDLINE, PubMed, CINAHL, and SPORTDiscus. The search was updated on October 2020. The first and main search process was conducted in the MEDLINE database using the appropriate Boolean operators and subject headings, as shown in Table 1. A similar search strategy was subsequently utilized to extract studies from other databases. The bibliographies of the pertinent studies were manually searched for eligible studies to eventually attain a comprehensive research process.

### 2.3. Data Collection and Extraction

The search results were uploaded to Mendeley software (Mendeley Ltd., London, UK). All duplicate results were removed. Screening of the studies was performed by two independent reviewers. Initially, titles were screened to select compatible studies. Abstracts and full texts were then analyzed to arrive at a list of eligible studies. Failure to reach a consensus regarding study inclusion was resolved by discussion and counseling with a third reviewer.

The extracted data included the basic criteria of each study (author names, year of publication, sample size, and study design), participants’ information (average age, weight, gender, and current involvement in a sports activity), kinematic variables, and strength measures of interest. Screening tools and functional tasks were also collected, as appropriate.

### 2.4. Types of Outcome Measures

The primary outcome of all included studies was to measure hip muscle strength and DKV, represented as the frontal plane projection angle, in healthy individuals. The statistical relationships between hip muscle strength and DKV were extracted, as appropriate. However, all included studies measured the association by using correlation coefficients (*r*). The strength of the correlation coefficients was interpreted and categorized following Zou et al. [20] into weak (*r* < 0.5), moderate (0.5 ≤ *r* < 0.8), and strong (*r* ≥ 0.8). A *p*-value less than 0.05 was considered statistically significant.

### 2.5. Assessment of Risk of Bias

The selected studies were evaluated using the Joanna Briggs Institute checklist for cross-sectional and case–control studies [21] (Supplementary Table S1). Each item on the scale was scored as “Yes,” “No,” “Not applicable,” or “Unclear.” A total quality score out of 8 and 10 for cross-sectional and case–control studies, respectively, was generated by assigning a score of 1 for each “Yes” received. Two authors independently assessed the quality of each study, while a third reviewer was consulted in case of any disagreement.

## 3. Results

### 3.1. Results of the Search Process

**Figure 1:**
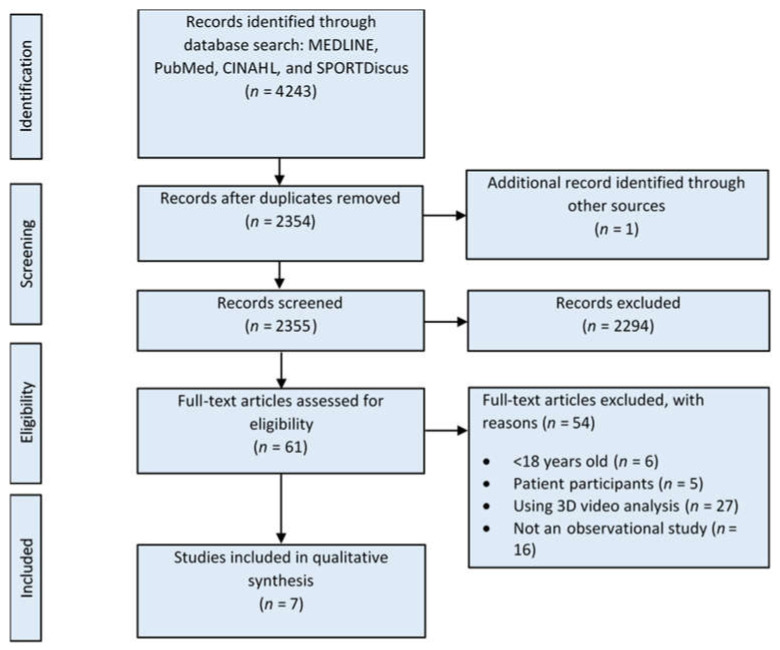
A flow diagram depicting the search process used in this review.

The initial search yielded 4243 studies retrieved from MEDLINE (*n* = 1468), PubMed (*n* = 1326), CINAHL (*n* = 595), and SPORTDiscus (*n* = 854). After screening the titles and abstracts of these studies, the full text of 61 articles was assessed for formal eligibility. Ultimately, seven studies were valid for inclusion in the final qualitative analysis. The PRISMA flow diagram summarizing the search results and screening workflow is shown in Figure 1.

### 3.2. Risk of Bias in the Included Studies

The total quality of cross-sectional studies assessed by the Joanna Briggs Institute appraisal checklist ranged from 5–7, whereas the total quality score of the one case–control study was 10 (Supplementary Table S1). All of the studies (*n* = 7) used reliable and valid outcomes to measure muscle strength and DKV, defined the inclusion criteria clearly, and used appropriate statistical analyses. However, only three studies accounted for the confounding variables. Furthermore, only two studies adequately reported the study settings and demographics of the participants.

### 3.3. Characteristics of the Included Studies

All included studies were cross-sectional. One study employed a case–control design, in which a group of participants diagnosed with patellofemoral pain was involved [22]. However, we considered only the healthy group “control” for inclusion in the current qualitative analysis. For the assessed muscle groups in the included studies, two studies measured hip abductor and external rotator strengths [23,24], while the remaining studies measured all muscle groups (hip abductors, extensors, and external rotators). The main characteristics of the included articles are listed in Table 2.

### 3.4. Characteristics of the Participants

A total of 277 participants were recruited in all studies, with females representing 53% (*n* = 146). Three studies investigated female-only cohorts [22,23,26]. Participants’ ages ranged from 18 to 45 years. All included studies recruited healthy participants (Table 2).

### 3.5. Measurement Methods of Hip Muscle Strength and Dynamic Knee Valgus

#### 3.5.1. Hip Strength Assessment

Five studies used handheld dynamometry (HHD) [22,23,26,27,29], one study used a Biodex System 3 Pro (Biodex Medical Systems, Inc., Shirley, New York, NY, USA) [28], and one study used a Biodex Isokinetic Dynamometer (Biodex Medical System, Inc., Shirley, New York, NY, USA) [25] to measure hip strength. All studies measured the isometric peak torque and normalized strength against the participant’s body mass.

Studies that used an HHD device to measure muscle strength also used a support strap to help resist the participant’s force. This method helps control the HHD device during muscle strength measurement, which helps mitigate the risk of bias of the examiner [30]. However, using hands to resist a participant’s force during measurement may eventually affect the accuracy of the results.

#### 3.5.2. Dynamic Knee Valgus Assessment

Two-dimensional video analysis using a digital camera and photo editing software, which has been found to be valid and reliable [18,29], was utilized for kinematic measurements of the knee valgus in all included studies. Further information on the cameras models and software used for the measurement of knee valgus in each study are displayed in Table 2.

### 3.6. Relationship between the Strength of Hip Muscles and Dynamic Knee Valgus

The relationship between the DKV and the strength of hip abductors and/or hip external rotators was investigated in all of the included studies and/or hip extensors in five studies [22,25,26,27,28]. The main outcome measures of the included studies, including the reported correlation between hip muscle strength and DKV, are shown in Table 3.

#### 3.6.1. Relationship between Hip Abductor Strength and Dynamic Knee Valgus

There was conflicting evidence from seven studies that investigated the relationship between hip abductor strength and DKV. Two studies revealed a weak–moderate significant negative correlation between hip abductor strength and DKV during a single-leg squat test [25] or single-limb step-down test [22]. However, another two studies revealed a weak positive correlation between hip abductor strength and DKV when participants performed single-limb step-down [23] or single-leg squat [26] tests. Furthermore, other studies found no statistically significant correlations during a single-leg squat test [24,28], a single-leg drop [28], or a forward lunge [27].

#### 3.6.2. Relationship between Hip Extensor Strength and Dynamic Knee Valgus

We found conflicting evidence from five studies that investigated the relationship between hip extensor strength and DKV. Two studies found a negative correlation, moderate [25] or weak [26], between hip extensor strength and DKV during a single-leg squat test. Conversely, no significant correlations were found in other studies between the two variables during step-down tests [22], single-leg squat tests [27], or forward lunges [28].

#### 3.6.3. Relationship between Hip External Rotator Strength and Dynamic Knee Valgus

The outcomes of these variables were conflicting. Two studies showed a weak positive correlation between hip external rotators and DKV in female participants during a single-leg squat test [24,26]. However, no relationship between these parameters was observed in cohorts comprising both males and females [25,27,28] or in cohorts that were exclusively females [22,23].

## 4. Discussion

This review aimed to synthesize the current evidence investigating the relationship between hip muscle strength (extensor, abductor, or external rotator) and DKV during dynamic tasks among healthy adults using a 2D analysis system. The results of this review found conflicting evidence suggesting that there is no clear relationship between hip muscle strength and DKV. Based on the results from seven studies, it was demonstrated that the relationship between hip abductor strength and DKV was negatively correlated in two studies, positively correlated in two studies, and not correlated in three studies. Meanwhile, based on the results from five studies, it was demonstrated that the relationship between hip extensor strength and DKV was negatively correlated in two studies and not correlated in three studies. Lastly, based on the results from seven studies, it was demonstrated that the relationship between hip external rotators and DKV was positively correlated in two studies and not correlated in five studies.

The findings of our review agree with the results of previous reviews published previously, in which that the relationship between hip muscle strength and DKV among studies is conflicting [10,11]. These conflicting results might be explained by the limitations identified in the included studies, which might have affected the interpretation of the results. Several methodological approaches have been used to evaluate hip muscle strength. There is no particular test for hip muscle strength regarded as the gold standard. In this review, five studies used HHD, which is inexpensive and easy to use, making it the most acceptable method for the measurement of hip muscle strength in clinical settings [31], although it has limitations. The examiner can experience difficulties keeping the dynamometer in the correct place while trying to stabilize the subject. This is particularly difficult if the examiner manually provides resistance. This method is also prone to inter-tester bias, as examiners can have varying levels of strength [32]. Therefore, these reasons may explain the conflicting results.

The meta-analysis conducted by Dix et al. [11] concluded that a reduction in the strength of hip extensors, external rotators, and abductors is associated with the DKV during single-leg drop landings, but not double-leg landings. They suggested that the distinction in the results of the kinematic evaluation of the lower limbs between single- and double-leg tasks is largely due to varying demands and muscle recruitments that require significant eccentric work to provide control in the frontal plane angles. In the included studies in our review, single-leg squat tests were frequently used (four studies out of seven). Such tests are utilized clinically as a screening approach for hip muscle dynamic control and the kinematics of the lower extremity, as they require adequate body control over a planted leg [33]. While using single-leg squats as the functional task, the isometric strength of hip extensors [25,26] is more likely to be correlated with the DKV than hip external rotators. The involvement of hip abductors can be explained by the lateral movement of the hip (to maintain body balance) during single-leg squat tasks [34], which increases the hip abduction angles and subsequently the DKV. For step-downs, the DKV was only correlated with hip abductor strength in healthy individuals [22,23]. Although hip movement in the non-stance leg is different for single-leg squats (backward rotation) and the step-down tests (forward rotation), hip abduction and flexion movements are similar in both tests [35].

The relevance of some strength measures to the functionality of hip muscles in certain sports is questionable. The nature of most physical activities is dynamic; therefore, it could be more appropriate to use dynamic or isotonic strength tests, rather than isometric tests. In this review, all of the included studies assessed the isometric strength of hip muscles, although the landing tasks required eccentric control in weight-bearing activities. This might affect the final outcomes and cause a lack of correlation between hip muscle strength and DKV in some studies. Furthermore, the participants’ positions in the included studies may not be optimal for the evaluation of the important hip muscles used to control hip kinematics. During landing tasks, hip muscle functionality may be better assessed in a closed-chain position on the stance leg. However, there is no reliable and valid method for testing hip strength in such position [30].

Task difficulty is another factor that may affect the interpretation of results. The results of this review warrant further investigation in terms of the functional tasks used. Employing difficult tasks would facilitate the discrimination of strength effects on lower limb kinematics by augmenting the challenge while controlling the DKV. Furthermore, difficult tests will allow for better applicability of the DKV in subjects with lower extremity injury compared to healthy populations. For the articles included in our review, both single-leg squat [36] and step-down [37] tasks were found to be sufficiently challenging for revealing kinematic differences. However, performing laboratory-based functional tasks seems to be different from performing the same tasks in a sporting context. The latter is frequently characterized by unpredictable movements in response to the movement of the ball or other players. For example, a previous study published by Mornieux et al. [38] showed that the hip joint plays a major role in increasing the frontal plane peak angles during unpredictable landing tasks.

It is also important to consider gender differences in knee kinematics during various functional tasks [39,40]. Of the five studies that recruited both males and females in the present review, none of them considered gender-based variations when measuring the correlation between hip muscle strength and DKV. However, the study conducted by Bin Hussein [25] found that females, compared to males, had weaker hip muscle strength and exhibited a greater peak knee valgus angle during a single-leg squat. A previous study also showed that female athletes have a 5.3 times higher risk of experiencing injuries relating to dynamic lower extremity valgus than their male counterparts [41]. Therefore, when combining the results of both male and female subjects, it is possible to mask some established correlated outcomes.

There are various methodological problems identified in the included studies, which should be considered when interpreting the findings of the current review. The majority of the included studies did not account for the confounding variables which might confound the association between hip muscle strength and DKV. Furthermore, only two studies adequately reported the study settings and demographics of the participants. Describing the study sample and study settings in sufficient detail can help other researchers to determine if it is comparable to other studies or to the population of interest to them. It was also observed that the sample sizes were relatively small in the included studies and the majority of these studies did not calculate the sample size using a power analysis method. Making mistakes in the calculation of the sample size might lead to insignificant or incorrect results [42,43]. Therefore, there is a need for future studies with sufficient sample sizes and with sufficient methodological quality addressing the limitations identified in the included studies in this review.

This review has several limitations. First, a potential bias may emerge due to the exclusion of non-English articles, because some significant articles might have escaped the search process. Second, the inclusion of different functional tasks would ultimately lead to considerable variation in strength challenges, which could impact the outcome. Third, the inclusion of female-only or mixed cohorts may render a difficult interpretation. Fourth, we were only able to include seven studies as very few studies met our eligibility criteria; therefore, more studies are needed.

## 5. Conclusion

The results regarding the relationship between peak the DKV and the isometric strength of hip muscles, represented as hip abductors, extensors, and external rotators, were conflicting. However, within the context of the limitations of the current literature, as well as the variation in methodological approaches, the clinical relevance of such findings should be interpreted cautiously. Therefore, it is recommended that future studies be conducted by measuring the eccentric strength of hip muscles, since this resembles muscular movement during landing. Furthermore, the functional tasks used to measure the DKV, such as cutting and maneuver tasks, should be more challenging.

## Figures and Tables

**Table 1:** Search strategy for the MEDLINE database.

Search	Query
1	Hip*
2	Results of item number 1 limited to English and human studies
3	Knee*
4	Results of item number 3 limited to English and human studies
5	Step down* OR drop vertical jump test OR single leg* OR single limb* OR drop jump* OR dynamic activities OR functional task OR leg drop OR leg jump OR jump* OR leg hop* OR hop*
6	Results of item number 3 limited to English and human studies
7	Results of item numbers 2, 4, and 6 were combined with “AND”

**Table 2:** The main characteristics of the included studies.

Author, Year	Study Design	Population	Sex	Age, Weight, Height	Hip Muscle Strength Measurement	Kinematic Measurement	Camera Model	Software
Almeida et al. [22], 2016	Cross-sectional case-control study	Healthy volunteers	21 F	18–45 (27.3 ± 4.5) years, 60.8 ± 7.3 kg, 1.63 ± 0.05 m	Isometric muscle torque using HHD	2D video analysis	Sony Cyber-shot DSC-W35	VirtualDub software (Copyright Avery Lee 1998–2009)
Bin Hussein [25], 2016	Cross-sectional study	General college population	17 F and 13 M	18–38 years, 68.3 ± 9.8 kg, 171.1 ± 7.2 cm	Isometric muscle testing using a Dillon EDjunior dynamometer, measured in kilogram–force (kgf)	2D video analysis	Sony DCRTRV19E (Japan)	SiliconCOACH Pro Version 6 (SiliconCOACH Ltd.; New Zealand)
Hollman et al. [23], 2009	Cross-sectional study	Healthy volunteers	20 F	24 ± 2.6 years, 66.4 ± 9.3 kg, 169.1 ± 9.4 cm	Maximum isometric force production capability using HHD	2D video analysis	Sony DCR-HC65 (Sony Corp. of America; New York, NY, USA)	DX9–Shareware version 2.6 software (The Rehabilitation Centre; Ottawa, ON, Canada)
Stickler et al. [26], 2015	Cross-sectional study	Healthy volunteers	40 F	18–30 (22.88 ± 0.32) years, 60.36 ± 1.7 kg, 165.5 ± 0.86 cm	Isometric “make” test using HHD used to assess peak force	2D video analysis	Sony Handycam DCR-HC37	Dartfish (Alpharetta, GA, USA)
Thijs et al. [27], 2007	Cross-sectional study	Healthy volunteers from a military academy	8 F and 76 M	18–30 years, average weight of 70.2 kg, average height of 177.7 cm	Isometric muscle testing using HHD with the examiner’s hand holding the dynamometer	2D video analysis	Sony HC20E camera (Sony Corp.; Tokyo, Japan)	Dartfish video software solutions (Fribourg, Switzerland)
Waldhelm et al. [28], 2017	Cross-sectional study	Healthy volunteers from a university population	18 F and 18 M	F: 21.0 ± 1.2 years, 69.4 ± 13.2 kg, 165.4 ± 8.4 cm M: 30.4 ± 6.4 years, 69.8 ± 9.2 kg, 1.7 ± 0.1 m	Isometric strength tests using Biodex System 3 Pro	2D video analysis	AIPTEK INC. (Irving, CA, USA)	CorrelDraw (Chicago, IL, USA)
Willson et al. [24], 2006	Cross-sectional study	Active athletes	22 F and 24 M	F: 19.4 ± 0.7 years, 66 ± 6.4 kg, 1.72 ± 0.07 m M: 19.9 ± 2.3 years, 79.8 ± 10.4 kg, 183 ± 0.10 m	Peak isometric torque using HHD	2D video analysis	Digital camera	CorrelDraw (Chicago, IL, USA)

Abbreviations: 2D, two-dimensional; F, female; M, male; HHD, handheld dynamometry.

**Table 3:** The main outcome measures of the included studies.

First Author, Year	Functional Tasks	Hip Muscle Group Measured	Relationship Between Hip Muscle Strength and Dynamic Knee Valgus
Almeida et al. [22], 2016	Single-leg step-down	Abductors	Weak negative correlation (*r* = −0.31, *p* = 0.047)
		Extensors	Nonsignificant correlation (*r* = −0.15, *p* > 0.05)
		External rotators	Nonsignificant correlation (*r* = −0.28, *p* > 0.05)
Bin Hussein [25], 2016	Single-leg squat	Abductors	Moderate negative correlation (*r* = −0.550, *p* = 0.002)
		Extensors	Moderate negative correlation (*r* = −0.421, *p* = 0.021)
		External rotators	Nonsignificant correlation (*r* = −0.206, *p* = 0.275)
Hollman et al. [23], 2009	Single-limb step down	Abductors	Weak positive correlation (*r* = 0.455, *p* = 0.022)
		External rotators	Nonsignificant correlation (*r* = 0.124, *p* > 0.05)
Stickler et al. [26], 2015	Single-leg squat	Abductors	Weak positive correlation (*r* = 0.466, *p* = 0.002)
		Extensors	Weak negative correlation (*r* = −0.396, *p* = 0.012)
		External rotators	Weak positive correlation (*r* = 0.464, *p* = 0.003)
Thijs et al. [27], 2007	Forward lunge	Abductors	Nonsignificant correlation (*r* = −0.002, *p* = 0.99)
		Extensors	Nonsignificant correlation (*r* = 0.11, *p* = 0.49)
		External rotators	Nonsignificant correlation (*r* = −0.05, *p* = 0.75)
Waldhelm et al. [28], 2017	Single-leg squat and single-leg drop	Abductors	Single-leg squat: Nonsignificant correlation (*r*^2^ = 0.002, *p* > 0.05) Single-leg drop: Nonsignificant correlation (*r*^2^ = 0.00004, *p* > 0.05)
		Extensors	Single-leg squat: Nonsignificant correlation (*r*^2^ = 0.006, *p* > 0.05) Single-leg drop: Nonsignificant correlation (*r*^2^ = 0.032, *p* > 0.05)
		External rotators	Single-leg squat: Nonsignificant correlation (*r*^2^ = 0.02, *p* > 0.05) Single-leg drop: Nonsignificant correlation (*r*^2^ = 0.003, *p* > 0.05)
Willson et al. [24], 2006	Single-leg squat	Abductors	Nonsignificant correlation (*r* = 0.23, *p* = 0.07)
		External rotators	Weak positive correlation (*r* = 0.4, *p* = 0.004)

## Data Availability

The data presented in this study are available from the corresponding author on reasonable request.

## Supplementary Materials

The following are available online at https://www.mdpi.com/article/10.3390/ijerph18147669/s1, Table S1: Quality assessment score of the included studies according to the Joanna Briggs Institute checklist.
