# Supplementary material for: Is Hip Muscle Strength Associated with Dynamic Knee Valgus in a Healthy Adult Population? A Systematic Review
Source: Int J Environ Res Public Health. 2021 Jul 19;18(14):7669. doi: 10.3390/ijerph18147669 (PMC8304771; doi:10.3390/ijerph18147669)
Supplement: Supplementary file 1 [file ijerph-18-07669-s001.zip › ijerph-1270447-supplementary.pdf]

Supplementary Table S1

a) Quality assessment score of the included studies according to the Joanna Briggs Institute checklist for cross-sectional studies.

First author	1	2	3	4	5	6	7	8	Total (Out of 8)
**Bin Hussein [1]**	Yes	Yes	Yes	Yes	Unclear	Unclear	Yes	Yes	**6**
**Hollman, et al. [2]**	Yes	No	Yes	Yes	No	No	Yes	Yes	**5**
**Stickler, et al. [3]**	Yes	No	Yes	Yes	Unclear	Unclear	Yes	Yes	**5**
**Thijs, et al. [4]**	Yes	No	Yes	Yes	Yes	Yes	Yes	Yes	**7**
**Waldhelm and Li [5]**	Yes	No	Yes	Yes	Unclear	Unclear	Yes	Yes	**5**
**Willson, et al. [6]**	Yes	No	Yes	Yes	Yes	Yes	Yes	Yes	**7**

The checklist items were as follows:

1. Were the criteria for inclusion in the sample clearly defined?
2. Were the study subjects and the setting described in detail?
3. Was the exposure measured in a valid and reliable way?
4. Were objective, standard criteria used for measurement of the condition?
5. Were confounding factors identified?
6. Were strategies to deal with confounding factors stated?
7. Were the outcomes measured in a valid and reliable way?
8. Was appropriate statistical analysis used?

Note: A total quality score out of eight was generated by assigning a score of one for each 'Yes' received.

b) Quality assessment score of the included studies according to the Joanna Briggs Institute checklist for case-control studies.

First author	1	2	3	4	5	6	7	8	9	10	Total (Out of 10)
**Almeida, et al. [7]**	Yes	Yes	Yes	Yes	Yes	Yes	Yes	Yes	Yes	Yes	**10**

The checklist items were as follows:

1. Were the groups comparable other than the presence of disease in cases or the absence of disease in controls?
2. Were cases and controls matched appropriately?
3. Were the same criteria used for identification of cases and controls?
4. Was exposure measured in a standard, valid and reliable way?
5. Was exposure measured in the same way for cases and controls?
6. Were confounding factors identified?
7. Were strategies to deal with confounding factors stated?
8. Were outcomes assessed in a standard, valid and reliable way for cases and controls?
9. Was the exposure period of interest long enough to be meaningful?
10. Was appropriate statistical analysis used?

Note: A total quality score out of 10 was generated by assigning a score of one for each 'Yes' received.

1. Bin Hussein, AS. "The Relationship between Hip Strength and Peak Knee Valgus Angle During Single Leg Squat." *Journal of Nursing and Health Science* 5, no. 4 (2016): 99-106.
2. Hollman, J. H., B. E. Ginos, J. Kozuchowski, A. S. Vaughn, D. A. Krause, and J. W. Youdas. "Relationships between Knee Valgus, Hip-Muscle Strength, and Hip-Muscle Recruitment During a Single-Limb Step-Down." *Journal of Sport Rehabilitation* 18, no. 1 (2009): 104-17.
3. Stickler, L., M. Finley, and H. Gulgin. "Relationship between Hip and Core Strength and Frontal Plane Alignment During a Single Leg Squat." *Physical Therapy in Sport* 16, no. 1 (2015): 66-71.
4. Thijs, Youri, Damien Van Tiggelen, Tine Willems, Dirk De Clercq, and Erik Witvrouw. "Relationship between Hip Strength and Frontal Plane Posture of the Knee During a Forward Lunge." *British Journal of Sports Medicine* 41, no. 11 (2007): 723-27.
5. Waldhelm, Andy, and Li Li. "Weak Relationships between Three Clinical Assessments and Core Stability Tests." *EC Orthopaedics* 7, no. 2 (2017): 88.
6. Willson, J. D., M. L. Ireland, and I. Davis. "Core Strength and Lower Extremity Alignment During Single Leg Squats." *Medicine and science in sports and exercise* 38, no. 5 (2006): 945-52.
7. Almeida, G. P., A. P. Silva, F. J. Franca, M. O. Magalhaes, T. N. Burke, and A. P. Marques. "Relationship between Frontal Plane Projection Angle of the Knee and Hip and Trunk Strength in Women with and without Patellofemoral Pain." *Journal of back and musculoskeletal rehabilitation* 29, no. 2 (2016): 259-66.
